# Healthy U.S.-style dietary patterns can be modified to provide increased energy from protein

**DOI:** 10.1186/s12937-022-00794-w

**Published:** 2022-06-18

**Authors:** Mary M. Murphy, Leila M. Barraj, Kelly A. Higgins

**Affiliations:** 1 Exponent, Inc., 1150 Connecticut Ave, NW, Washington, DC 20036 USA; 2 USDA-ARS, BHNRC, Food Components and Health Lab, 10300 Baltimore Avenue, RM. 231, BG. 307B, BARC-EAST, Beltsville, MD 20705 USA

**Keywords:** Dietary patterns, Protein foods, Processed meat, NHANES, Sodium reduction

## Abstract

**Background:**

Dietary patterns developed by the USDA provide modest levels of protein (14–18% energy) within the Acceptable Macronutrient Distribution Range (AMDR) of 10–35% for adults, though diets providing a higher percentage of energy may be beneficial for some individuals. The purpose of this study was to determine if it is feasible to modify the Healthy U.S.-Style Eating Pattern (“HEP”) to provide a higher percentage of energy from protein.

**Methods:**

Using the framework implemented by the USDA in developing the HEP, energy from protein was set at 20%, 25%, and 30%. Amounts of protein foods were proportionally increased while amounts of other foods were adjusted iteratively within specified parameters. The models also disaggregated total meat/poultry into fresh and processed forms to develop patterns maintaining current proportions, current levels, reduced, or no processed meat/poultry. Nutrient intakes were compared with nutrient goals for representative U.S. populations with 2,000 kcal needs (females 19–30 years, males 51–70 years), with 90% of the Recommended Dietary Allowance or Adequate Intake regarded as sufficient.

**Results:**

Dietary patterns with 20% energy from protein were constructed with minor deviations from the current 2,000 kcal HEP. Dietary patterns with 25% energy from protein were constructed for all levels of processed meat/poultry excluding the current proportion model, though relative to the current HEP the constructed patterns reflect substantial reductions in amounts of refined grains and starchy vegetables, and substantial increases in protein foods consumed as beans and peas, seafood, and soy products. It was not possible to develop a pattern with 30% energy from protein without reducing the percentage of energy from carbohydrate below the AMDR or non-compliance with other modeling constraints. Stepwise reductions in processed meat/poultry reduced sodium intake.

**Conclusions:**

It is feasible to develop dietary patterns in a 2,000 kcal diet while mirroring the HEP that meet recommended intakes of nutrients with 20% or 25% energy from protein, though the pattern with 25% energy from protein may be more idealistic than realistic. Reduced levels of processed meat/poultry may translate to lower sodium intake.

## Introduction

The Dietary Guidelines for Americans (DGA) identify several healthy dietary patterns that Americans can follow to meet nutrient needs within energy requirements, including the Healthy U.S.-Style (“HEP”), the Healthy Mediterranean-Style (“MEP”), and the Healthy Vegetarian (“VEP”) Eating Patterns [1]. These food patterns identify recommended intakes of key food groups, subgroups, and components to meet nutrient needs and DGA recommendations across a range of calorie levels [1]. The food patterns from USDA were designed to meet nutrient recommendations established in the Dietary Reference Intakes (DRIs), including macronutrient levels that fall within the Acceptable Macronutrient Distribution Ranges (AMDR) [2]. For an adult, the AMDR for carbohydrate is 45–65% of energy and the AMDR for fat is 20–35% of energy. The balance, 10–35% of energy, represents the AMDR for protein. In a 2,000 kcal diet, the model HEP and MEP patterns provide 18% of energy from protein and the VEP provides 14% of energy from protein, which are levels in the lower to middle range of the protein AMDR.

Dietary sources of protein in the HEP include a variety of plant and animal-based foods, including lean meats, poultry, eggs, seafood, nuts, seeds, and soy products that collectively account for nearly 40% of total dietary protein. Dairy foods contribute nearly 30% of total dietary protein, while the balance is provided by grains, vegetables, and fruits [3]. The DGA as well as guidance from the American Heart Association support consumption of protein from a variety of animal and plant sources, which reflects the preferences of many Americans as assessed in nationwide surveys [1, 4]. Americans are, however, encouraged to limit consumption of red meat and in particular processed meat [1], largely based on concerns of chronic disease risk observed in epidemiological studies [5–8]. Some evidence suggests that adverse effects of meat intake may be attributed to processed meat rather than total meat [9, 10], and evidence indicates that effects vary based on the comparator diet [11].

Randomized controlled studies provide evidence that dietary patterns delivering a higher proportion of energy as protein, including higher protein as meat, may in fact support health. For example, compared with a balanced diet proving 17% energy as protein and 33% energy as fat (12% energy as saturated fat), a diet providing 27% energy as protein and 28% energy as fat (6% energy as saturated fat) with 153 g lean beef daily for five weeks resulted in lower total cholesterol and LDL-cholesterol and lower systolic blood pressure in a sample of adults with hypercholesterolemia [12, 13]. Dietary patterns providing a higher percentage of energy from protein while remaining within the AMDR (i.e., no more than 35% energy from protein) may have beneficial effects including improved body weight management in adults with overweight or obesity [14], or lean mass and handgrip strength in the elderly when combined with resistance exercise [15]. Findings from a 2021 meta-analysis of 54 randomized controlled trials show that compared to diets providing on average 18% energy from protein (range of 10–23%), higher protein diets (average of 28% energy from protein, range of 20–45%) can support weight loss and reductions in fat mass, and lower systolic blood pressure, total cholesterol, triacylglycerol, and fasting insulin without adverse effects on other cardiometabolic risk factors [16]. This meta-analysis did not include an assessment based on protein source due to the absence of source information in most studies [16], though other research indicates that different sources of protein may differentially affect health outcomes, with neither plant nor animal sources consistently identified as superior [17, 18]. Dietary patterns providing a higher percentage of energy as protein therefore may be of interest for some individuals, though alternate dietary patterns must of course meet all nutrient needs to ensure nutritional balance.

The purpose of this study was to determine if it is feasible to modify the 2,000 kcal HEP (i.e., M-HEP) to provide a higher percentage of energy from protein while meeting all nutrient needs using the framework implemented by the USDA in developing the HEP. The models also disaggregated total meat/poultry into fresh and processed forms under scenarios representing processed meat/poultry at the current proportion of total meat/poultry representative of typical consumption among the U.S. population, the current level (i.e., no increase in ounce-eq/week of processed meat/poultry despite increased intake of protein foods), a reduced level of processed meat/poultry, and no processed meat/poultry. This study provides an exploratory step in considering dietary patterns with a higher percentage of energy from both animal and plant sources represented by the protein foods group and subgroups in the DGA, including meat/poultry, seafood, eggs, nuts, seeds, and soy products.

## Materials and methods

### Overview and data inputs

This modeling study is based on the framework and data developed by USDA for food pattern modeling. USDA established an approach for food pattern modeling to develop dietary patterns that meet nutrient goals for the U.S. population ages 2 years and older [19]. The approach has been used to develop the HEP, and also the first food patterns for infants and toddlers introduced in the 2020–2025 DGA [3, 19–21]. The established method for developing dietary patterns is a 5-step process defined as follows: Step 1) establish energy levels, Step 2) establish nutritional goals, Step 3) establish food groupings and food group amounts, Step 4) determine nutrients obtained from foods within each group, and Step 5) evaluate nutrient levels compared to nutrient goals. The underlying data used by USDA in the development of dietary patterns for the 2020–2025 DGA are two days of food intake as reported in the National Health and Nutrition Examination Survey (NHANES) 2015–2016, and the Standard Reference (SR) Legacy nutrient composition data used by USDA to process nutrient intakes in the survey. In the current study, modifications were made only to the percentage of energy from macronutrients necessary to achieve the objective of the study (step 2 of the 5-step process), and these modifications in turn required revision of food group amounts (step 3 of the 5-step process) to reflect the adjustment in macronutrient goals.

### Nutritional goals and modifications to macronutrient distributions

The defining characteristic of the patterns modeled in this study is a higher level of energy from protein relative to the HEP. In this study, the modified HEP models with higher protein are referred to as “M-HEP”. Modified patterns were constructed with 20% (M20-HEP), 25% (M25-HEP), and 30% (M30-HEP) energy from protein, or 100 g, 125 g, and 150 g protein, respectively, in a 2,000 kcal diet. Food Patterns developed by USDA represent 12 energy levels from 1,000 to 3,200 kcal to address the range of energy needs for most individuals ages 2 years and older. Given the exploratory nature of this study, patterns were developed for the 2,000 kcal diet as a representative pattern. The 2,000 kcal diet is appropriate for energy needs of adolescents and adults over a range of ages and activity levels, including older sedentary men and older active women. Consistent with the approach used in development of dietary patterns in the DGA, nutrient requirements for patterns at the 2,000 kcal energy recommendation were designed to meet the DRIs for females ages 19–30 years and males ages 51–70 years [22].

### Modifications to the protein food group amounts and fresh vs processed forms

The nutrient profiles for all food groups and subgroups used in this modeling are the consumption-weighted nutrient-dense food averages for the U.S. population ages 2 years and older as established by the food pattern modeling team for the 2020–2025 HEP [22]. Recommended amounts of foods from each food group in the HEP for a 2,000 kcal diet provided the starting values for all food groups and subgroups in these models, and consumption-weighted data for the U.S. population ages 2 years and older were selected for representative nutrient profiles of all food groups for the U.S. population. The HEP was modified to meet the nutrient goals of 20% (M20-HEP), 25% (M25-HEP), and 30% (M30-HEP) energy from protein by adjusting contributions of protein foods to meet the protein targets. The constructed diets largely align with the HEP, with modifications necessary only to provide higher levels of protein while still meeting nutrient goals.

The protein subgroups modified to develop the HEP in this analysis are subgroups for meat, poultry, eggs, seafood (with separate groups for seafood with higher and lower concentrations of omega-3 fatty acids, i.e., high n-3 and low n-3), nuts/seeds, and soy products. The beans and peas group, which counts towards either the vegetable or protein foods group in USDA food patterns, was also a potential source of protein in the models. Consistent with the DGA, in this study the meat group (referenced in some literature as red meat [23]) includes beef, pork, lamb, goat, and game meat, all in either unprocessed (fresh) or processed forms, while the poultry group includes chicken, turkey, Cornish hens, duck, goose, and game birds, likewise all in unprocessed (fresh) or processed forms. Lean meat or poultry therefore includes both fresh and processed forms.

In USDA’s development of the HEP, the nutrient profile of each food group represents a composite of the population weighted average nutrient profiles of similar foods in the constituent subgroups referred to as item clusters. Representative data selected by USDA for the nutrient profiles typically reflect nutrient data for a food with the least amount of added sugars, sodium, and solid fat. Examples of item clusters for protein foods include lean meat, poultry and fish cooked without added fat or salt, canned fish without added salt, hard-boiled whole eggs, reduced-fat hot dogs, reduced-fat turkey sausage (representative of all sausage), unsalted nuts, tofu, and soy protein isolate.

In the HEP, item clusters for meat and poultry include fresh and processed (i.e., cured) forms of these protein foods. For the current study, item clusters corresponding to fresh and processed forms of meat/poultry were disaggregated into the proportions of ounce-equivalents (ounce-eq) used by USDA in development of the 2020–2025 HEP (Table 1). The meat and poultry groups combined account for 68% of all ounce-eq in the protein foods group, with 57% as meat and 43% as poultry. Approximately one-third (34%) of all meat is represented by processed meat, while 12% of all poultry is represented by processed poultry. Across the combined subgroups of meat and poultry, 24% of these protein foods are represented by processed products and the balance (76%) is represented by fresh products. The proportionally weighted nutrient profiles for the disaggregated fresh and processed item clusters within the meat and poultry subgroups developed for use in this analysis, along with the nutrient profiles for all protein foods subgroups, are shown in Table 2. All values in Table 2 represent the nutrient profile per 1 ounce-eq of these protein foods, defined as 1 ounce lean meat, poultry, or seafood, 1 egg, ¼ cup cooked beans or tofu, 1 tablespoon peanut butter, or ½ ounce nuts/seeds.

**Table 1:** Contributions of fresh and processed meat and poultry item clusters to protein foods in the HEP

**Item clusters in the red meat and poultry food subgroups**^a^	**Representative Food**	**Contribution to Protein Foods (%)**	**Contribution to Food Subgroup (%)**
**Red meat**		**38.21**	**100**
**Fresh**			
Beef	Beef, round, eye of round, separable lean only, roasted	9.06	23.71
Beef, ground	Ground beef, 97% lean, patty, pan-broiled	10.36	27.12
Game meat	Deer, top round, lean only, steak, broiled	0.35	0.91
Lamb	Lamb, domestic, leg, separable lean only, choice, roasted	0.32	0.83
Liver	Beef, liver, pan-fried	0.23	0.61
Pork, fresh	Pork, fresh, sirloin chops, boneless, lean, broiled	4.88	12.76
*Subtotal*		*25.2*	*65.94*
**Processed**			
Sausage	Turkey sausage, reduced fat, brown and serve, cooked	2.53	6.62
Luncheon meats, beef	Frankfurter, beef, low fat	3.35	8.75
Luncheon meats, pork	Ham, sliced, extra lean	4.72	12.34
Pork, cured	Pork, cured, ham, whole, separable lean only, roasted	2.42	6.35
*Subtotal*		*13.02*	*34.06*
**Poultry**		**29.39**	**100**
**Fresh**			
Chicken	Chicken, meat only, roasted	24.37	82.9
Turkey	Turkey, meat only, roasted	1.6	5.44
*Subtotal*		*25.97*	*88.34*
**Processed**			
Luncheon meats, poultry	Turkey breast, sliced, prepackaged	3.43	11.66
*Subtotal*		*3.43*	*11.66*
**Total red meat and poultry**		**67.62**	
*Fresh*		*51.17*	*76*
*Processed*		*16.45*	*24*

^a^Contributions calculated from data used by USDA in the development of the U.S. Healthy Style Eating Pattern (HEP) [22]. Consistent with the DGA, the meat group in this study includes beef, pork, lamb, goat, and game meat, all in either unprocessed (fresh) or processed forms, while the poultry group includes chicken, turkey, Cornish hens, duck, goose, and game birds, likewise all in unprocessed (fresh) or processed forms. Lean meat or poultry therefore includes both fresh and processed forms

**Table 2:** Consumption-weighted average nutrient profiles per ounce-eq for meat and poultry, including fresh and processed forms

	**Meat**			**Poultry**			**Fish**		**Eggs**	**Soy Products**	**Nuts/ Seeds**	**Beans and Peas**
	**Total**	**Fresh**^**a**^	**Processed**^**a**^	**Total**	**Fresh**^**a**^	**Processed**^**a**^	**Hi n3**	**Lo n3**				
**Macronutrients**												
Calories, kcal	43.57	44.59	40.73	50.98	53.32	30.05	55.73	32.49	77.50	47.65	88.37	60.67
Protein, g	6.94	7.93	4.87	7.79	8.20	4.20	6.47	6.41	6.29	11.81	3.07	3.96
Carbohydrate, g	0.28	0.01	0.79	0.07	0.00	0.62	0.00	0.17	0.56	0.19	3.29	10.82
Fiber, dietary, g	0.01	0.00	0.02	0.00	0.00	0.00	0.00	0.00	0.00	0.06	1.11	3.79
Total lipid (fat), g	1.46	1.19	1.97	1.94	2.04	1.07	3.11	0.54	5.31	0.70	7.68	0.35
Saturated fats, g	0.48	0.47	0.49	0.53	0.56	0.26	0.64	0.15	1.63	0.11	1.15	0.07
Linoleic acid, g	0.12	0.06	0.22	0.37	0.38	0.25	0.18	0.05	0.59	0.28	1.99	0.10
Linolenic acid, g	0.01	0.00	0.01	0.02	0.02	0.01	0.03	0.01	0.02	0.04	0.14	0.06
Cholesterol, mg	20.07	23.37	13.23	24.27	25.44	13.89	18.12	25.00	186.50	0.00	0.00	0.00
**Minerals**												
Calcium, mg	2.47	2.09	3.17	4.22	4.22	3.97	7.76	10.16	25.00	35.77	29.85	19.52
Iron, mg	0.55	0.68	0.29	0.32	0.34	0.12	0.15	0.24	0.60	1.95	0.44	1.05
Magnesium, mg	6.12	6.59	5.07	7.00	7.16	5.39	9.29	9.00	5.00	7.38	30.34	23.62
Phosphorus, mg	63.61	64.40	60.83	57.86	55.75	70.59	74.53	73.54	86.00	106.46	60.74	66.96
Potassium, mg	92.94	96.03	85.06	73.53	68.82	105.18	110.00	83.04	63.00	19.98	93.70	180.78
Sodium, mg	99.63	16.16	261.11	51.61	24.64	254.58	22.97	97.95	62.00	128.40	7.60	0.69
Zinc, mg	1.15	1.44	0.56	0.57	0.60	0.27	0.14	0.42	0.53	0.57	0.48	0.47
Copper, mg	0.05	0.06	0.02	0.02	0.02	0.01	0.02	0.06	0.01	0.22	0.13	0.10
Selenium, μg	8.04	8.62	6.75	6.12	6.39	3.69	12.72	14.39	15.40	0.75	1.53	1.60
**Vitamins**												
Vitamin A, μg RAE	13.83	20.88	0.11	3.85	4.33	0.00	17.33	7.41	74.50	0.00	0.05	0.03
Vitamin E, mg AT	0.07	0.07	0.06	0.07	0.07	0.04	0.30	0.27	0.52	0.00	1.82	0.25
Vitamin D, IU	3.76	2.05	7.05	1.54	1.50	1.70	133.76	40.70	43.50	0.00	3.47	0.00
Vitamin C, mg	0.03	0.00	0.07	0.00	0.00	0.00	0.80	0.22	0.00	0.01	0.05	0.30
Thiamin, mg	0.06	0.05	0.08	0.02	0.02	0.01	0.08	0.01	0.03	0.03	0.03	0.09
Riboflavin, mg	0.06	0.07	0.06	0.05	0.05	0.04	0.04	0.02	0.26	0.02	0.05	0.03
Niacin, mg	1.63	1.87	1.14	2.56	2.61	2.03	2.09	1.30	0.03	0.19	1.30	0.20
Vitamin B-6, mg	0.11	0.13	0.09	0.13	0.14	0.12	0.16	0.06	0.06	0.02	0.06	0.07
Vitamin B-12, μg	0.58	0.77	0.19	0.10	0.10	0.10	0.86	1.13	0.56	0.00	0.00	0.00
Choline, mg	26.01	28.79	20.07	21.04	22.52	8.53	20.51	19.03	146.90	26.08	8.71	12.77
Vitamin K, μg	0.30	0.38	0.13	0.57	0.64	0.00	0.05	0.12	0.15	0.16	0.60	1.58
Folate, μg DFE	2.36	2.64	1.77	1.69	1.75	1.13	7.60	3.95	22.00	23.59	11.97	68.47

^a^Contributions calculated from data used by USDA in the development of the U.S. Healthy Style Eating Pattern (HEP) [22]

### Analysis

A series of food pattern models was developed using stepwise reductions in the number of ounce-eq of processed meat/poultry relative to levels in the HEP. At each target level of energy from protein (i.e., 20%, 25%, and 30%), a pattern that maintained the HEP ratio of processed to fresh meat and poultry ounce-eq (i.e., 34:66 for processed: fresh meat and 12:88 for processed: fresh poultry) and proportionately increased all protein foods was first attempted. Patterns that proportionately increased all protein foods other than processed meat and poultry were then modeled, thus maintaining current allowances for processed meat and processed poultry in the HEP, namely 4.5 and 1.0 ounce-eq per week, respectively, based on weighted consumption data for the U.S. population. Contributions of processed meat and poultry were then decreased to 2 and 0.5 ounce-eq per week, respectively; an additional model was developed in which all processed meat/poultry was eliminated. If the M-HEP could not be achieved with proportional increases in all protein food subgroups, the subgroups were individually adjusted. With the exception of eggs, minimum levels of weekly ounce-eq from each protein subgroup were maintained at levels no lower than levels in the HEP. The weekly allowance for eggs, which are a concentrated source of cholesterol, was reduced to offset the cholesterol provided by additional ounce-eq of meat, poultry, and seafood.

To accommodate increased energy from protein foods, levels of refined grains and starchy vegetables were first decreased and energy allowed from the solid fats and added sugars components of the “calories for other uses” was reduced. To maintain some flexibility in other dietary choices and consistency with the HEP, the M-HEP retained total calories for other uses in the range of 75% to 100% of calories allocated for this use in the HEP (i.e., approximately 180 to 242 kcal). The percent allocation of solid fat and added sugars was maintained approximately in the ratio used in the HEP of 16 g solid fat and 27 g added sugars.

In developing the M-HEP in this study, nutrient goals consistent with those used by the USDA were used to assess nutrient adequacy. Nutrient goals for the patterns are diets within the macronutrient AMDRs, at least 90% of the Recommended Dietary Allowance (RDA) or Adequate Intake (AI) for micronutrients, fiber, and fatty acids as established by the IOM [2, 24, 25], below the Chronic Disease Risk Reduction (CDRR) for sodium [25], and within quantitative recommendations in the 2020–2025 DGA for saturated fat [1]. In the current analysis, M-HEP model diets likewise were considered nutritionally sufficient if the levels of nutrients were at least 90% of the RDA/AI for both males ages 51–70 years and females ages 19–30 years which are representative populations consuming a 2,000 kcal diet and the nutrient goals used by USDA to assess compliance [22]. The HEP developed by USDA does not meet the specified nutrient goals for vitamin D, vitamin E, choline, iron (females) and magnesium (males) [22]. If the level of these nutrients in a model was marginally less than the specified RDA/AI target (i.e., 90% of the RDA/AI), the model was considered sufficient, which is consistent with the approach utilized by the USDA [20]. Although the 2020–2025 DGA does not specify a limit on dietary cholesterol, Americans are encouraged to consume only as much as necessary within a nutritionally adequate diet [1]. The patterns modelled in this study were designed to provide less than 300 mg cholesterol, which is the limit used in food pattern modeling exercises to support the 2020–2025 DGA [22].

In developing the M-HEP, food group recommendations for underconsumed foods were maintained within the median and 95th percentiles of Usual Intakes (UI) to maintain feasible dietary patterns while overconsumed components were maintained between 5^th^ percentile and median intakes [22]. Given the exploratory nature of this modeling exercise, this constraint was not consistently enforced and deviations are noted.

The patterns generated following this stepwise approach were further reviewed and modified by manually adjusting food group amounts to create food patterns translatable for communicating dietary guidance, which is consistent with the refinements detailed by USDA in development of the HEP. These modifications included rounding food groups to the nearest half unit (e.g., 2.4 units was rounded to 2.5 units) and minor adjustments to minimize variations within a food group across food patterns for a given protein level.

## Results

The modified HEP diets (at 2,000 kcal) designed to meet the target levels of energy from protein and target allowances of processed meat and poultry are summarized in Table 3. Nutrient profiles for these food patterns and a comparison of the nutrient content of each pattern to reference nutrient goals are provided in Table 4.

**Table 3:** Modified HEP with 20% or 25% energy from protein and varying proportions of processed meat/poultry

		**HEP**^**a**^	**M20-HEP**^**b**^				**M25-HEP**^**b**^		
		**Scenarios for Proportion of Total Meat/Poultry as Processed Meat/Poultry**							
**Food Group and Subgroup**	**Intake Frequency**	**Current %**	**Current %**	**Current Amount**	**Reduced Amount**	**None**	**Current Amount**	**Reduced Amount**	**None**
**Total fruit**	**ounce-eq/ day**	**2**	**2**	**2**	**2**	**2**	**2**	**2**	**2**
**Total vegetables**	**ounce-eq/ week**	**17.5**	**17.5**	**17.5**	**17.5**	**17.5**	**19**	**19**	**19**
Dark green		1.5	1.5	1.5	1.5	1.5	1.5	1.5	1.5
Red-orange		5.5	5.5	5.5	5.5	5.5	5.5	5.5	5.5
Beans and peas		1.5	1.5	1.5	1.5	1.5	5	5	5
Starchy		5	5	5	5	5	3	3	3
Other		4	4	4	4	4	4	4	4
**Total grains**	**ounce-eq/ day**	**6**	**5.5**	**5.5**	**5.5**	**5.5**	**4**	**4**	**4**
Whole grains		3	3	3	3	3	3	3	3
Refined grains		3	2.5	2.5	2.5	2.5	1	1	1
**Total protein foods**	**ounce-eq/ week**	**39**	**46.5**	**46.5**	**46.5**	**44.0**	**64.0**	**64.5**	**64.0**
**Meats, poultry, eggs**	**ounce-eq/** **week**	**26**	**31**	**31**	**31**	**29.5**	**41.5**	**42**	**41.5**
Meats^*c*^		12.5	15	15	15	14.5	21.5	22	21.5
*Meats, fresh*		*8.5*	10	11	13	14.5	17.5	19.5	21.5
*Meats, processed*		*4.5*	5	4.5	2	0	4.5	2	0
Poultry^*c*^		10.5	12.5	12.5	12.5	12	18	18	18
*Poultry, fresh*		*9.5*	11	11.5	12	12	17	17.5	18
*Poultry processed*		*1*	1.5	1	0.5	0	1	0.5	0
Eggs		3	3.5	3.5	3.5	3	2	2	2
**Total seafood**	**ounce-eq/ week**	**8**	**10**	**10**	**10**	**9.5**	**14.5**	**14.5**	**14.5**
Fish-Hi n3		2.1	2.5	2.5	2.5	2.4	3.6	3.6	3.6
Fish-Lo n3		6.3	7.6	7.6	7.6	7.2	10.8	10.9	10.7
**Nuts, seeds, soy products**	**ounce-eq/ week**	**5**	**5.5**	**5.5**	**5.5**	**5**	**8**	**8**	**8**
Soy products		0.5	0.5	0.5	0.5	0.5	4	4	4
Nuts/seeds		4	5	5	5	4.5	4	4	4
**Total dairy**	**cup-eq/ day**	**3**	**3**	**3**	**3**	**3**	**3**	**3**	**3**
Oils	g/day	27	27	27	27	27	27	27	27
Total energy other sources	kcal/day	242	226	222	222	242	181	181	181
Solid *fats*	g/day	16	15	15	15	16	13	13	13
Added sugars	g/day	27	25	24	24	27	18	18	18

^a^Healthy U.S. Style Eating Pattern (HEP)

^b^Modified Healthy U.S. Style Eating Pattern (M-HEP) with 20% energy from protein (M20-HEP) or 25% energy from protein (M25-HEP). Processed meat was modeled under four scenarios: (1) maintain current proportion of total meat/poultry as processed; (2) maintain the current level of total meat/poultry as processed (i.e., ounce-eq/week of processed meat/poultry consistent with level in the HEP), (3) reduce the level of total meat/poultry as processed (i.e., approximately one-half the ounce-eq of processed meat/poultry consistent in the HEP), and (4) no processed meat/poultry. In all scenarios, the level of fresh meat/poultry was calculated as the difference between total meat/poultry and the level allocated to processed forms

^c^Contributions calculated from data used by USDA in the development of the (HEP) [22]. Consistent with the DGA, the meat group in this study includes beef, pork, lamb, goat, and game meat, all in either unprocessed (fresh) or processed forms, while the poultry group includes chicken, turkey, Cornish hens, duck, goose, and game birds, all in unprocessed (fresh) or processed forms

**Table 4:** Nutrient intakes and comparisons to nutrient goals for the M20-HEP and M25-HEP models

			**HEP**^**a**^			**M20-HEP**^**b**^			**M25-HEP**^**b**^		
**Nutrient**	**Measure**	**Reference Intakes by Population**^**c**^		**% of Reference**			**% of Reference**			**% of Reference**	
**Macronutrients**		**F 19–30 y; M 51–70 y**	**Intake**	**F** **19–30 y**	**M** **51–70 y**	**Intake**	**F** **19–30 y**	**M** **51–70 y**	**Intake**	**F** **19–30 y**	**M** **51–70 y**
Calories, kcal	%goal	2000	2001	100%	100%	2001–2003	100%	100%	2002–2005	100%	100%
Protein, g	%RDA	46; 56	92	200%	164%	98–100	213%-217%	175%-179%	125–126	272%-274%	223%-225%
	%kcal	10–35		18%	18%		20%	20%		25%	25%
Carbohydrate, g	%RDA	130	259	198%	198%	248–250	191%-192%	191%-192%	228	175%	175%
	%kcal	45–65		52%	52%		50%	50%		46%	46%
Fiber, dietary, g	%AI	28	30	108%	108%	30	107%	107%	35	125%	125%
Total lipid (fat), g	%kcal	20–35	71	32%	32%	72–73	32%-33%	32%-33%	70	31%	31%
Saturated fats, g	%kcal	< 10	18	8%	8%	18	8%	8%	17	8%	8%
Linoleic acid, g	%AI	12; 14	20	164%	141%	20	167%	143%	19	158%	136%
Linolenic acid, g	%AI	1.1; 1.6	2.3	212%	146%	2.4	218%	150%	2.4	218%	150%
Cholesterol, mg	mg	< 300	214	75%	75%	241–255	80%-85%	80%-85%	266–272	89%-90%	89%-90%
**Minerals**											
Calcium, mg	%RDA	1000; 1200	1278	128%	107%	1272–1277	127%-128%	106%	1295–1296	130%	108%
Iron, mg	%RDA	18; 8	14	79%	178%	14	78%	175%	16–17	89%-94%	200%-213%
Magnesium, mg	%RDA	310; 420	358	116%	86%	363–366	117%-118%	86%-87%	407	131%	97%
Phosphorus, mg	%RDA	700	1654	237%	237%	1686–1714	241%-245%	241%-245%	1933–1938	276%-277%	276%-277%
Potassium, mg	%AI	2600; 3400	3390	131%	100%	3442–3477	132%-134%	101%-102%	3791–3797	146%	112%
Sodium, mg	%CDRR	2300	1658	72%	72%	1443–1680	63%-73%	63%-73%	1405–1597	65%-69%	65%-69%
Zinc, mg	%RDA	8; 11	13	162%	118%	14	175%	127%	16–17	200%-213%	145%-155%
Copper, mg	%RDA	0.9	1.4	155%	155%	1.4	156%	156%	1.7	189%	189%
Selenium, μg	%RDA	55	113	205%	205%	118–120	215%-218%	215%-218%	132–133	240%	240%
**Vitamins**											
Vitamin A, μg RAE	%RDA	700; 900	898	129%	100%	909–918	130%-131%	101%-102%	908–920	130%-131%	101%-102%
Vitamin E, mg AT	%RDA	15	10	70%	70%	11	71%	71%	11	72%	72%
Vitamin D, IU	%RDA	600	300	52%	52%	314–325	52%-54%	52%-54%	350–354	59%	59%
Vitamin C, mg	%RDA	75; 90	129	172%	144%	129	172%	143%	126	168%	140%
Thiamin, mg	%RDA	1.1; 1.2	1.8	166%	152%	1.7	155%	142%	1.7	155%	142%
Riboflavin, mg	%RDA	1.1; 1.3	2	183%	154%	2	182%	154%	2	182%	154%
Niacin, mg	%RDA	14; 16	23	164%	144%	25	179%	156%	27–28	193%	169%
Vitamin B-6, mg	%RDA	1.3; 1.7	2.2	167%	128%	2.3	177%	135%	2.6	200%	153%
Vitamin B-12, μg	%RDA	2.4	6.2	260%	260%	6.7–7.0	279%-292%	279%-292%	8.1–8.4	338%-342%	338%-342%
Choline, mg	%AI	425; 550	355	86%	67%	380–393	89%-92%	69%-71%	441–446	104%	80%-81%
Vitamin K, μg	%AI	90; 120	140	155%	117%	141–142	157%-158%	118%	142–143	158%	118%
Folate, μg DFE	%RDA	400	513	129%	129%	495–498	124%-125%	124%-125%	573	143%	143%

^a^Healthy U.S. Style Eating Pattern (HEP); sums may not add up to 100% due to rounding

^b^Modified Healthy U.S. Style Eating Pattern (M-HEP) with 20% energy from protein (M20-HEP) or 25% energy from protein (M25-HEP); sums may not add up to 100% due to rounding

^c^Nutrient Goals for representative populations with a 2,000 kcal energy needs (females ages 19–30 y [F19-30 y], and males ages 51–70 y [M51-70 y]): Acceptable Macronutrient Distribution Ranges (AMDR) for macronutrients, Recommended Dietary Allowances (RDA) and Adequate Intakes (AI) [2, 24, 25], the 2020–2025 DGA limit for energy from saturated fat [1], the Chronic Disease Risk Reduction (CDRR) level for sodium [25], and the cholesterol limit used in food pattern modeling exercises to support the 2020–2025 DGA[22]

### Modified food groups with 20% energy from protein

It was feasible to develop a M20-HEP (i.e., 100 g protein daily) that proportionately increased all protein foods, and a M20-HEP that proportionately increased all protein foods while maintaining processed meat/poultry at the current proportion, current level, at a reduced level, or eliminating all processed meat/poultry. The model diets provide 50% energy as carbohydrate and 32-33% energy as fat (Table 4). Development of the M20-HEP patterns was achieved with a reduction of 0.5 ounce-eq per day refined grains and elimination of 16–20 kcal from the calories for other uses group (i.e., elimination of up to 1 g solid fat and 2–3 g added sugars) for models including processed meat/poultry. In the M20-HEP that eliminated processed meat/poultry, no reduction in calories for other uses was necessary and protein needs were met with 44 ounce-eq per week of protein foods compared to 46.5 ounce-eq per week in models with processed meat/poultry.

### Modified food groups with 25% energy from protein

Under the specified constraints of this study, it was not feasible to develop a M25-HEP (i.e., 125 g protein daily) that proportionately increased all protein foods while maintaining the HEP proportion of protein foods as processed meat/poultry. It was, however, feasible to develop M25-HEP patterns that increased protein foods while maintaining processed meat/poultry at the current level, at a reduced level, or eliminating all processed meat/poultry. These diets provide 46% energy as carbohydrate and 31% energy as fat (Table 4). M25-HEP models reflecting current amounts, reduced amounts, or no processed meat/poultry were constructed by limiting increases in the eggs subgroup (2 eggs per week) to offset additional sources of cholesterol, maintaining HEP levels of nuts and seeds (4 ounce-eq per week), increasing soy products to 4 ounce-eq per week and increasing beans and peas by 3.5 cup eq/week (i.e., 14 ounce-eq per week), and proportionally increasing meat, poultry, and seafood protein foods to meet the protein target. Refined grains were reduced from 3 to 1 ounce-eq per day and starchy vegetables were reduced from 5 to 3 ounce-eq per week. In all models, 62 kcal were eliminated from the category of calories for other uses through reduction of 3 g solid fat and 9 g added sugars. Total weekly protein foods amounts are 64 to 64.5 ounce-eq in all models.

### Modified food groups with 30% energy from protein

It was not possible to develop a diet providing 30% of energy from protein (i.e., 150 g protein daily) under the constraints specified for this study, specifically a diet satisfying the AMDR with at least 45% energy from carbohydrate while maintaining a balance of solid fat and added sugars in the calories for other uses category.

### Modifications to overconsumed nutrients in the modified patterns

**Fig. 1:**
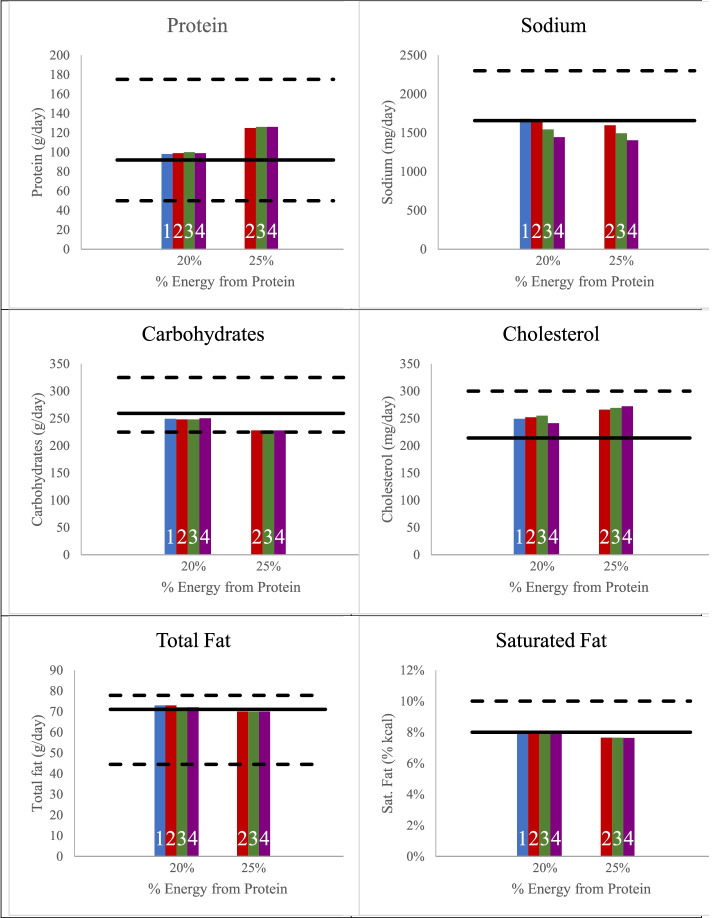
Intake of macronutrients and nutrients to limit in the M20-HEP and M25-HEP models. The solid black line represents the nutrient concentration per 2,000 kcal provided by the Healthy U.S.-Style Eating Pattern (HEP) and the dashed black lines represent the Acceptable Macronutrient Distribution Ranges (AMDR) for macronutrient intake by adults as established by the IOM,^2^ the Chronic Disease Risk Reduction (CDRR) level for sodium [25], and the cholesterol limit used in food pattern modeling exercises to support the 2020–2025 DGA [22]. Blue bars (1) are patterns with the same proportion of processed meat/poultry as the HEP (34% of total meat and 12% of total poultry as processed); red bars (2) are patterns with the current level of processed meat (4 ounce-eq per week) / poultry (1.5 ounce-eq per week); green bars (3) are patterns with a reduced level of processed meat (2 ounce-eq per week) / (1 ounce-eq per week) poultry; and purple bars (4) are patterns with no processed meat/poultry

In both the M20-HEP and M25-HEP models, the stepwise reductions in weekly ounce-eq of processed meat/poultry resulted in reductions in daily sodium intake. As shown in Fig. 1, sodium declined in the M20-HEP from 1,680 mg in the pattern with the current proportion of processed meat/poultry (6.5 ounce-eq per week) to 1,647 mg, 1,542 mg, and 1,443 mg in the modified patterns with the current level (5.5 ounce-eq per week), reduced level (3 ounce-eq per week), and no processed meat/poultry, respectively. Likewise, stepwise reductions in processed meat/poultry in the M25-HEP model resulted in reductions in sodium from 1,597 mg in the pattern with the current level of processed meat/poultry to 1,494 mg and 1,405 mg in the modified patterns with reduced (3 ounce-eq) and no processed meat/poultry patterns, respectively.

The M20-HEP and M25-HEP models provide no more than 8% energy from saturated fat (Fig. 1). In contrast to sodium and saturated fat, the level of cholesterol in the model diets was consistently higher relative to the level of 214 mg in the HEP, with cholesterol ranging from 241 to 255 mg in the M20-HEP, and from 266 to 272 mg in the M25-HEP.

### Achievement of nutrient goals in the modified patterns

Per the constraints of the study, the model patterns developed for M20-HEP and M25-HEP meet or exceed nutrient goals for all nutrients for which HEP met at least 90% of the RDA/AI (Table 4). Consistent with the USDA’s HEP, the M20-HEP and M25-HEP also do not meet nutrient goals for vitamin E and vitamin D. For vitamin E, the modified plans provide 71–72% of the RDA (vs. 70% in the HEP), while for vitamin D the M20-HEP and M25-HEP provide 52–54% and 59% of the RDA, respectively (vs. 52% in the HEP). With the exception of the M20-HEP with no processed meat/poultry, which provides 89% of the choline AI for females ages 19–30 years, all modified patterns meet or exceed the choline goals for females ages 19–30 years. For males ages 51–70 years, the M20-HEP provide 69–71% of the AI and the M25-HEP provide 80–81% of the AI for choline. Consistent with the HEP, some of the dietary patterns provide less than 90% of the RDA for magnesium among adult males. The M20-HEP provide 86–87% of the RDA for magnesium for adult males ages 51–70 years, which is consistent with the level provided by the HEP for this population (86%), while the M25-HEP provide 97% of the magnesium RDA for these males, which meets the nutrient goal. For females ages 19–30 years, the M20-HEP and the M25-HEP provide a minimum of 78% and 89%, respectively, of the RDA for iron, thus all models are comparable to or higher than the 79% of the iron RDA provided by the HEP.

### Comparison of higher protein patterns to usual intake

Dietary patterns that are within 5^th^ and 50^th^ percentile of UI for over-consumed foods and the 50^th^ and 95^th^ percentile of UI for under-consumed food could not be achieved for the M25-HEP. In all M25-HEP scenarios, refined grains are below the 5^th^ percentile UI of 2.4 g ounce-eq/day, while beans and peas are above the 95^th^ percentile (5.5 vs. 5 cup-eq/week), total seafood exceeds the 95^th^ percentile of UI of 11.9 ounce-eq/week, and soy products exceeds the 95^th^ percentile of UI of 2.8 ounce-eq/week. For the M25-HEP, solid fats are less than the 5^th^ percentile of 15 g/day (Table 5).

**Table 5:** Food group amounts in M20-HEP and M25-HEP vs 5^th^ and 95^th^ percentiles of usual intake

Pattern	Basis for Amount of Processed Meat/Poultry^a^	Food Group Recommendation < 5th Percentile	Food Group Recommendation > 95th Percentile
M20-HEP	Current %, Current amount, Reduced amount, None	• No deviations^b^	• No deviations^b^
M25-HEP	Current %	-^c^	-
	Current amount, Reduced amount, None	• Refined grains • Solid fats	• Beans and peas • Total seafood • Soy products

^a^Current %, current proportion of processed meat/poultry in HEP; Current amount, current level of processed meat/poultry in HEP; Reduced amount, reduced level of processed meat/poultry compared to HEP; None, no processed meat/poultry

^b^Only deviations beyond those present within the HEP [22] are noted here

^c^Pattern not feasible in this modeling study

## Discussion

This modeling study demonstrates that the Healthy U.S. Style Eating Pattern (HEP), which provides 18% energy from protein, can be modified to provide 20% or 25% energy from protein while meeting nutrient needs in a 2,000 kcal diet. Under the constraints of the models in this study, however, it was not feasible to construct a pattern with 30% energy from protein without reducing the percentage of energy from carbohydrate below the carbohydrate AMDR and remaining compliant with other nutrient constraints. In the M20-HEP models, increased energy from proportional increases in all protein foods was offset by reductions in refined grains with little to no reduction in calories from other sources required to meet nutrient needs. In the M25-HEP models, further reductions in refined grains, reductions in starchy vegetables, and shifts in protein foods including reductions in eggs, increases in beans and peas, and proportionately higher increases in soy products, along with greater reductions in energy designated for other uses, were required to meet the protein goal and nutrient needs. Stepwise reductions in levels of processed meat and poultry in the M20-HEP and M25-HEP models were accompanied by reductions in sodium intake while elimination of processed products in the M20-HEP allowed for a small reduction in total protein foods to meet the protein target.

We are aware of a previous study in which dietary pattens providing a higher percentage of energy from protein were constructed; in that study, models providing 18.8% and 30% energy as protein were developed, primarily by using nonessential calories for additional protein [26]. In the current study, patterns maintained at least 75% of calories for other uses (i.e., approximately 180 or more of the 242 kcal) to allow for consumer choice and ideally diets to which individuals can adhere. Under these constraints, it was not feasible to develop a 30% energy from protein pattern. In contrast to the current study, the study by Wolfe and colleagues did not manipulate contributions of fresh vs. processed meat and poultry.

Dietary guidance recommends limiting intake of processed meat/poultry [1]. Nonetheless, food pattern models in the DGA, which reflect consumption patterns by the U.S. population, assume approximately one-quarter of all meat and poultry combined is consumed in a processed form, and approximately one-third of all meat is a processed meat [22, 27]. In developing the dietary patterns in this study, protein food subcategories of meat and poultry were disaggregated into weighted proportions of fresh and processed components. This disaggregation of fresh and processed meat and fresh and processed poultry components allowed the models to examine the effects of stepwise reductions in the levels of processed products on nutrient intakes, which is a major strength of this study. Processed meat/poultry including cold cuts and cured meats and bacon, frankfurters, and sausages are among the top sources of sodium in the U.S. diet [28], and most Americans are encouraged to decrease intake of sodium to support cardiovascular health [4]. As shown in the stepwise reductions of processed meat/poultry in both the M20-HEP and M25-HEP models, elimination of processed products may translate to reductions in sodium on the order of 200–300 mg daily.

The models in this study demonstrate that consumption of fresh forms of lean meat and poultry, or select forms of other protein foods, can more efficiently meet protein needs as processed forms provide relatively low levels of protein per ounce-eq. While higher fat meats may be a source of saturated fat in the diet, lean meats are a modest source of this component. Per ounce-eq, processed meat provides more total fat than fresh meat, though the saturated fat content is comparable at 0.49 and 0.47 g, respectively. No increase in overall total fat intake or saturated fat intake (or percent energy from saturated fat) was observed, which is a reflection of the nutrient profiles of the foods in the modified patterns and the reduction of 1–3 g solid fat in the models.

In all modeled dietary patterns, review of the nutrient intakes and comparisons to reference intakes demonstrate that nutrient intakes are comparable or, in some cases, enhanced relative to the HEP. The level of dietary choline, for example, is adequate for females in the modified diets while it is below recommended levels in the HEP. This shift may be attributed to an increase in eggs in the M20-HEP and the increase in beans and peas in the M25-HEP. In all models, ounce-eq of refined grains are reduced to provide a key source of increased energy from protein. While many refined grains are enriched and make important nutrient contributions to the diet, including the micronutrients folate, iron, and magnesium [29], reduced levels of refined grains do not have a detrimental effect on nutrient intakes, likely because these micronutrients are present across a range of protein foods and in particular bean and peas. The M-HEP do provide higher levels of cholesterol (an additional 27–41 mg per day for M20-HEP, and 52–58 mg per day for M25-HEP), though all models remain below the limit of 300 mg per day used in this analysis. A 2020 science advisory from the American Heart Association concluded that dietary patterns emphasizing lean protein sources and other foods naturally low in cholesterol, and providing cholesterol levels comparable or lower than current intake in the U.S., are recommended for cardiovascular health [30]. Overall the M-HEP provide nutritionally adequate patterns comparable to the USDA HEP.

The models developed in this study demonstrate that a 2,000 kcal diet with 20% energy from protein can be achieved with minor modifications to the HEP, namely reduction of 0.5 ounce-eq refined grains daily and use of no more than 20 kcal from the calories for other uses category in models with some processed meat/poultry. With the elimination of processed meat/poultry, the modified protein diet was constructed without compromising any calories for other uses. For individuals interested in slightly increasing energy from protein while eliminating processed meat from the diet, the M20-HEP with no processed meat provides a feasible pattern. Models with 25% energy from protein were constructed in this exercise, though with greater restrictions, including more substantial reductions in amounts of refined grains as well as reductions in starchy vegetables and some substantial modifications in the specific forms of protein foods consumed, namely in the weekly ounce-eq of beans and peas, seafood, and soy products. It is noteworthy that the levels of beans and peas and the level of seafood in this pattern are comparable to levels in the MEP or VEP in a 2,000 kcal diet. Nonetheless, it may be difficult for individuals interested in consuming more energy as protein to easily transition to these dietary patterns while staying within the recommended energy levels given substantial shifts in amounts of some food groups, thus the patterns may be more idealistic than realistic for some individuals.

The current modeling exercise did not consider impact on the environment, though this issue warrants consideration in future research on dietary patterns. The M-HEP models in this analysis were developed assuming proportionally higher intake of all foods within the protein foods group defined in the DGA, which include both animal and plant protein sources. Reduced consumption of animal-based foods is considered a strategy to support environmental sustainability [3, 31]. The relationship between optimal dietary patterns and environmental sustainability is, however, complex, as a recent systematic review of dietary patterns in the U.S. recommends that future research also consider the water demands of higher consumption of plant-based foods and nutritional tradeoffs associated with dietary shifts away from animal sources [31].

Strengths of this study include use of the USDA food pattern modeling methods to develop higher protein patterns based largely on the HEP. Protein foods were increased in proportions aligning with proportions in the HEP in the M20-HEP, and as feasible in the M25-HEP. A significant feature of this study is the disaggregation of the meat and poultry subgroups into fresh and processed components, which allows for modeling to develop diets with reduced or no processed meat/poultry. In future analyses of dietary intakes, disaggregation of the fresh and processed meat/poultry components will be important for understanding sources of nutrients and effects of different food selections.

The study is a modeling exercise and not without limits. As with all food pattern modeling, the diets developed in these patterns represent diets composed of foods with little to no added sodium, sugar, or solid fat, and a specified allowance of calories as added sugars and solid fat for consumers to enjoy food forms that deviate from the reference foods or to consume additional foods. In reality, many individuals do not select diets completely aligned with the patterns and are not able to meet nutrient needs within energy needs. Further nutrition education is necessary to help consumers select nutrient-dense choices. While numerous constraints were developed to ensure that the resulting eating patterns would provide higher energy as protein while remaining generally similar to the HEP, it was not feasible to maintain alignment with the HEP in developing the M25-HEP. The patterns presented in this study represent possible dietary patterns for a 2,000 kcal diet, though numerous patterns could be developed with slightly different selections and constraints. In this exploratory study, patterns were constructed only for a 2,000 kcal diet. Additional modeling could be completed to cover the range of energy needs for most adults (i.e., 1,600 kcal to 3,200 kcal). It is noteworthy that the current HEP corresponding to 1,600 kcal provides 21% of energy as protein and the 1,800 kcal diet provides 19% of energy as protein [22]. Thus, relative to the 2,000 kcal diet, fewer if any modifications may be needed to achieve higher protein levels in the lower energy diets consumed by older adults or adults on weight loss diets for whom the patterns may be beneficial. Only foods in the protein foods (including beans and peas in the M25-HEP) were used to increase protein; other foods that are sources of protein such as dairy products were not modified as these are not recognized in the protein foods group in USDA food pattern modelling. Additionally, this study was designed only to examine the feasibility of constructing food patterns with higher energy from protein, not the health effects of these diets. Further analyses to determine the feasibility of higher protein diets based on other USDA patterns, i.e., the MEP and the VEP, are warranted to provide insight on other options.

## Conclusions

In summary, the models developed in this study demonstrate that it is feasible to meet recommended intakes of nutrients with consumption of diets similar to the HEP in a 2,000 kcal diet though modified to provide a higher proportion of energy from protein. Diets with 20% energy from protein were constructed with minor deviations from the current HEP. While nutritionally adequate diets with 25% energy from protein were constructed under the imposed constraints to maintain parallels to the HEP, the resulting diets may be regarded as considerably different from current dietary patterns, which could present challenges for adherence. It was not possible to develop a pattern with 30% energy from protein without reducing the percent energy from carbohydrate below the AMDR or non-compliance with other modeling constraints. Lastly, the disaggregation of meat and poultry into fresh and processed components serves to clearly demonstrate the effects of reducing levels of processed products in the diet, particularly on sodium consumption. Routinely presenting meat and poultry in terms of fresh and processed components may be helpful in educating consumers on the value of fresh meat/poultry while limiting consumption of processed meat/poultry.

## Data Availability

The data described in the article and used in the analysis are publicly available from the USDA as cited by the 2020 Dietary Guidelines Advisory Committee and Food Pattern Modeling Team: https://www.dietaryguidelines.gov/2020-advisory-committee-report/food-pattern-modeling.

## Abbreviations

AI: Adequate Intake
AMDR: Acceptable Macronutrient Distribution Range
CDRR: Chronic Disease Risk Reduction
cup-eq: Cup equivalent
DGA: Dietary Guidelines for Americans
DRI: Dietary Reference Intakes
HEP: Healthy U.S.-Style Eating Pattern
IOM: Institute of Medicine
M20-HEP: Modified Healthy U.S.-Style Eating Pattern—20% energy from protein
M25-HEP: Modified Healthy U.S.-Style Eating Pattern—25% energy from protein
M30-HEP: Modified Healthy U.S.-Style Eating Pattern—30% energy from protein
MEP: Healthy Mediterranean-Style Eating Pattern
M-HEP: Modified Healthy U.S.-Style Eating Pattern
NHANES: National Health and Nutrition Examination Survey
ounce-eq: Ounce equivalent
RDA: Recommended Dietary Allowance
SR: Standard Reference
UI: Usual Intake
USDA: United States Department of Agriculture
VEP: Healthy Vegetarian Eating Pattern

## References

[1] U.S. Department of Health and Human Services and U.S. Department of Agriculture: 2020–2025 Dietary Guidelines for Americans. 9th edition. 2020. Available at DietaryGuidelines.gov.

[2] Institute of Medicine (2006). Dietary reference intakes: the essential guide to nutrient requirements.

[3] Dietary Guidelines Advisory Committee (2015). Scientific Report of the 2015 Dietary Guidelines Advisory Committee: Advisory Report to the Secretary of Agriculture and the Secretary of Health and Human Services.

[4] Lichtenstein AH, Appel LJ, Vadiveloo M, Hu FB, Kris-Etherton PM, Rebholz CM, Sacks FM, Thorndike AN, Van Horn L, Wylie-Rosett J (2021). 2021 Dietary Guidance to Improve Cardiovascular Health: A Scientific Statement From the American Heart Association. Circulation.

[5] Bouvard V, Loomis D, Guyton KZ, Grosse Y, Ghissassi FE, Benbrahim-Tallaa L, Guha N, Mattock H, Straif K (2015). International Agency for Research on Cancer Monograph Working G: Carcinogenicity of consumption of red and processed meat. Lancet Oncol.

[6] Wang X, Lin X, Ouyang YY, Liu J, Zhao G, Pan A, Hu FB (2016). Red and processed meat consumption and mortality: dose-response meta-analysis of prospective cohort studies. Public Health Nutr.

[7] Micha R, Michas G, Mozaffarian D (2012). Unprocessed red and processed meats and risk of coronary artery disease and type 2 diabetes–an updated review of the evidence. Curr Atheroscler Rep.

[8] Pan A, Sun Q, Bernstein AM, Schulze MB, Manson JE, Willett WC, Hu FB (2011). Red meat consumption and risk of type 2 diabetes: 3 cohorts of US adults and an updated meta-analysis. Am J Clin Nutr.

[9] Abete I, Romaguera D, Vieira AR (2014). Lopez de Munain A, Norat T: Association between total, processed, red and white meat consumption and all-cause, CVD and IHD mortality: a meta-analysis of cohort studies. Br J Nutr.

[10] Larsson SC, Orsini N (2014). Red meat and processed meat consumption and all-cause mortality: a meta-analysis. Am J Epidemiol.

[11] Guasch-Ferre M, Satija A, Blondin SA, Janiszewski M, Emlen E, O’Connor LE, Campbell WW, Hu FB, Willett WC, Stampfer MJ (2019). Meta-Analysis of Randomized Controlled Trials of Red Meat Consumption in Comparison With Various Comparison Diets on Cardiovascular Risk Factors. Circulation.

[12] Roussell MA, Hill AM, Gaugler TL, West SG, Ulbrecht JS, Vanden Heuvel JP, Gillies PJ, Kris-Etherton PM (2014). Effects of a DASH-like diet containing lean beef on vascular health. J Hum Hypertens.

[13] Roussell MA, Hill AM, Gaugler TL, West SG, Heuvel JP, Alaupovic P, Gillies PJ, Kris-Etherton PM (2012). Beef in an Optimal Lean Diet study: effects on lipids, lipoproteins, and apolipoproteins. Am J Clin Nutr.

[14] Hansen TT, Astrup A, Sjodin A (2021). Are Dietary Proteins the Key to Successful Body Weight Management? A Systematic Review and Meta-Analysis of Studies Assessing Body Weight Outcomes after Interventions with Increased Dietary Protein. Nutrients.

[15] Kirwan RP, Mazidi M, Garcia CR, Lane KE, Jafari A, Butler T (2022). Protein interventions augment the effect of resistance exercise on appendicular lean mass and handgrip strength in older adults: a systematic review and meta-analysis of randomized controlled trials. Am J Clin Nutr.

[16] Vogtschmidt YD, Raben A, Faber I, de Wilde C, Lovegrove JA, Givens DI, Pfeiffer AFH, Soedamah-Muthu SS (2021). Is protein the forgotten ingredient: Effects of higher compared to lower protein diets on cardiometabolic risk factors. A systematic review and meta-analysis of randomised controlled trials. Atherosclerosis.

[17] Lim MT, Pan BJ, Toh DWK, Sutanto CN, Kim JE (2021). Animal protein versus plant protein in supporting lean mass and muscle strength: A systematic review and meta-analysis of randomized controlled trials. Nutrients.

[18] van Baak MA, Larsen TM, Jebb SA, Martinez A, Saris WHM, Handjieva-Darlenska T, Kafatos A, Pfeiffer AFH, Kunesova M, Astrup A (2017). Dietary Intake of Protein from Different Sources and Weight Regain, Changes in Body Composition and Cardiometabolic Risk Factors after Weight Loss: The DIOGenes Study. Nutrients.

[19] Britten P, Marcoe K, Yamini S, Davis C (2006). Development of food intake patterns for the MyPyramid Food Guidance System. J Nutr Educ Behav.

[20] Dietary Guidelines Advisory Committee (2020). Scientific Report of the 2020 Dietary Guidelines Advisory Committee: Advisory Report to the Secretary of Agriculture and the Secretary of Health and Human Services.

[21] Dewey KG, Pannucci T, Casavale KO, Davis TA, Donovan SM, Kleinman RE, Taveras EM, Bailey RL, Novotny R, Schneeman BO (2021). Development of Food Pattern Recommendations for Infants and Toddlers 6–24 Months of Age to Support the Dietary Guidelines for Americans, 2020–2025. J Nutr.

[22] Dietary Guidelines Advisory Committee and Food Pattern Modeling Team (2020). Food Pattern Modeling: Ages 2 Years and Older.

[23] O’Connor LE, Gifford CL, Woerner DR, Sharp JL, Belk KE, Campbell WW (2020). Dietary Meat Categories and Descriptions in Chronic Disease Research Are Substantively Different within and between Experimental and Observational Studies: A Systematic Review and Landscape Analysis. Adv Nutr.

[24] Institute of Medicine (2011). Dietary Reference Intakes for Calcium and Vitamin D.

[25] National Academies of Sciences (2019). Engineering, and Medicine: Dietary Reference Intakes for Sodium and Potassium.

[26] Wolfe RR, Cifelli AM, Kostas G, Kim IY (2017). Optimizing Protein Intake in Adults: Interpretation and Application of the Recommended Dietary Allowance Compared with the Acceptable Macronutrient Distribution Range. Adv Nutr.

[27] O’Connor LE, Wambogo EA, Herrick KA, Parsons R, Reedy J (2022). A standardized assessment of processed red meat and processed poultry intake in the US population aged ≥2 years using NHANES. J Nutr..

[28] Quader ZS, Zhao L, Gillespie C, Cogswell ME, Terry AL, Moshfegh A, Rhodes D (2017). Sodium Intake Among Persons Aged >/=2 Years - United States, 2013–2014. MMWR Morb Mortal Wkly Rep.

[29] Papanikolaou Y, Fulgoni VLI (2021). The Role of Fortified and Enriched Refined Grains in the US Dietary Pattern: A NHANES 2009–2016 Modeling Analysis to Examine Nutrient Adequacy. Front Nutr.

[30] Carson JAS, Lichtenstein AH, Anderson CAM, Appel LJ, Kris-Etherton PM, Meyer KA, Petersen K, Polonsky T, Van Horn L, American Heart Association Nutrition Committee of the Council on L (2020). Dietary Cholesterol and Cardiovascular Risk: A Science Advisory From the American Heart Association. Circulation.

[31] Reinhardt SL, Boehm R, Blackstone NT, El-Abbadi NH, McNally Brandow JS, Taylor SF, DeLonge MS (2020). Systematic Review of Dietary Patterns and Sustainability in the United States. Adv Nutr.

